# Bipolar Resistive Switching Characteristics of HfO_2_/TiO_2_/HfO_2_ Trilayer-Structure RRAM Devices on Pt and TiN-Coated Substrates Fabricated by Atomic Layer Deposition

**DOI:** 10.1186/s11671-017-2164-z

**Published:** 2017-06-08

**Authors:** Wei Zhang, Ji-Zhou Kong, Zheng-Yi Cao, Ai-Dong Li, Lai-Guo Wang, Lin Zhu, Xin Li, Yan-Qiang Cao, Di Wu

**Affiliations:** 1 National Laboratory of Solid State Microstructures, Materials Science and Engineering Department, College of Engineering and Applied Sciences, Collaborative Innovation Center of Advanced Microstructures, Nanjing University, Nanjing, 210093 People’s Republic of China; 2 Anhui Key Laboratory of Functional Coordination Compounds, School of Chemistry and Chemical Engineering, Anqing Normal University, Anhui, 246011 People’s Republic of China

**Keywords:** Atomic layer deposition, Resistive random access memory, Bottom electrode, Resistive switching parameters, Oxygen vacancy concentration, Trilayer structure

## Abstract

The HfO_2_/TiO_2_/HfO_2_ trilayer-structure resistive random access memory (RRAM) devices have been fabricated on Pt- and TiN-coated Si substrates with Pt top electrodes by atomic layer deposition (ALD). The effect of the bottom electrodes of Pt and TiN on the resistive switching properties of trilayer-structure units has been investigated. Both Pt/HfO_2_/TiO_2_/HfO_2_/Pt and Pt/HfO_2_/TiO_2_/HfO_2_/TiN exhibit typical bipolar resistive switching behavior. The dominant conduction mechanisms in low and high resistance states (LRS and HRS) of both memory cells are Ohmic behavior and space-charge-limited current, respectively. It is found that the bottom electrodes of Pt and TiN have great influence on the electroforming polarity preference, ratio of high and low resistance, and dispersion of the operating voltages of trilayer-structure memory cells. Compared to using symmetric Pt top/bottom electrodes, the RRAM cells using asymmetric Pt top/TiN bottom electrodes show smaller negative forming voltage of −3.7 V, relatively narrow distribution of the set/reset voltages and lower ratio of high and low resistances of 10^2^. The electrode-dependent electroforming polarity can be interpreted by considering electrodes’ chemical activity with oxygen, the related reactions at anode, and the nonuniform distribution of oxygen vacancy concentration in trilayer-structure of HfO_2_/TiO_2_/HfO_2_ on Pt- and TiN-coated Si. Moreover, for Pt/HfO_2_/TiO_2_/HfO_2_/TiN devices, the TiN electrode as oxygen reservoir plays an important role in reducing forming voltage and improving uniformity of resistive switching parameters.

## Background

Resistive random access memory (RRAM) has attracted great attention due to its potential for the replacement of flash memory in next-generation nonvolatile memories [1–3]. Resistive switching (RS) phenomenon has been widely discovered in transition metal oxides, solid electrolytes, and organic polymers [4–7]. RRAM devices based on transition metal oxides have been extensively explored because of its simple composition and compatible processing with conventional complementary metal-oxide-semiconductor (CMOS) fabrication [8–10]. The filament model of oxygen vacancy migration is used to elucidate the switching behavior [1, 11]. A unified microscopic principle is proposed to quantify both unipolar and bipolar switching characteristics of transition metal oxide-based RRAM, which are correlated with the distribution of localized oxygen vacancies in the oxide switching layer [12, 13].

However, memory cells using transition metal oxides suffer from nonuniformity of resistive switching parameters, such as unstable resistance values of low and high resistance states (LRS and HRS), dispersed set and reset voltages, impeding the commercial applications. Recently, the trilayer-structure oxide-based RRAM devices have been demonstrated to improve the dispersion of resistive switching parameters. The cells with a structure of Al_2_O_3_/HfO_2_/Al_2_O_3_ exhibited fantastic uniformity of set and reset voltages, and excellent endurance of switching between the LRS and HRS [14]. The linkage or rupture of the conductive filaments more easily occurred in two interfacial layers between Al_2_O_3_/IL/HfO_2_/IL/Al_2_O_3_. Meanwhile, the unit of trilayer-structure of TaO_x_/TiO_2_/TaO_x_ showed good performance in one selector-one resistor arrays, which was ascribed to the fact that the energy band of the TiO_2_ film was symmetrically bent at the top and bottom TaO_*x*_/TiO_2_ interfaces and modified as a crested oxide barrier due to some Ta atoms diffusion into TiO_2_ film [15].

Moreover, the RS behavior of a given oxide storage medium can be significantly affected by the electrode materials [1, 16, 17]. However, the existing models based on the free energy of interfacial oxide formation and the metal work functions are insufficient to completely explain the results. Meanwhile, the work on the electrodes dependent RS phenomenon of the trilayer-structure RRAM is also rather lacking at present.

Atomic layer deposition (ALD) is a new type of thin film deposition technology based on sequential self-limited and complementary surface chemisorptions reactions using precursor vapor with simple and precision thickness control, large area uniformity, and excellent three-dimensional conformality, especially for deposition of nano-laminated structure [18, 19].

In this work, the HfO_2_/TiO_2_/HfO_2_ trilayer-structure RRAM devices have been prepared on Si/SiO_2_/Ti/Pt and Si/TiN substrates with Pt top electrodes by ALD. The impact of the bottom electrodes of Pt and TiN on the RS behaviors of HfO_2_/TiO_2_/HfO_2_ devices has been investigated carefully. The related explanation has been proposed.

## Methods

In this experiment, we used two different bottom electrodes, including commercial Si/SiO_2_/Ti/Pt and home-made Si/SiO_2_/TiN. Conductive TiN was deposited by plasma-enhanced atomic layer deposition (PEALD) in our laboratory.

ALD was performed in a commercial Picosun SUNALE^TM^R-200 advanced reactor (Picosun, Finland). *P* type Si (100) wafers with a resistivity of 1~10 Ω cm were used as the starting substrates. After the conventional RCA cleaning of the Si wafers without removing native oxide, 30-nm-thick TiN was deposited on Si as the bottom electrode at 400 °C by PEALD, using room temperature TiCl_4_ and NH_3_ plasma gas as the Ti and N precursors, respectively. Liquid NH_3_ was selected as NH_3_ plasma source at room temperature. The plasma power and NH_3_ gas flow rate were 2500 W and 150 sccm, respectively.

Subsequently, 5 nm HfO_2_/10 nm TiO_2_/5 nm HfO_2_ stacking structures were deposited in turn on Pt- and TiN-coated Si substrates at 250 °C by thermal ALD using Hf[N(C_2_H_5_)CH_3_]_4_ (TEMAH), TiCl_4_, and H_2_O as the Hf, Ti, and O precursors, respectively, where one oxide cycle consisted of 0.1*s* metal source injection, 4*s* N_2_ purging, 0.1*s* H_2_O injection, and 4*s* N_2_ purging. TEMAH was evaporated at 150 °C. Pure N_2_ (99.999%) was used as carrier gas and purging gas. Then, 100-nm-thick Pt top electrodes were DC sputtered through a shadow mask with a diameter of 150 μm using the Q150T system.

The growth per cycle (GPC) of pure HfO_2_ or TiO_2_ on Si was determined by spectroscopic ellipsometer (GES-5, Sopra). The topography and surface roughness of the films and bottom electrodes were analyzed by atomic force microscopy (AFM, Cypher, Asylum Research). The root-mean-square (RMS) roughness values were recorded from 1 μm × 1 μm areas. The composition and chemical state of the stack structures were examined by X-ray photoelectron spectroscopy (XPS, Thermo Fisher K-Alpha) with a monochromatic Al K_α_ source (hν = 1486.6 eV) for excitation of photoelectrons. The charge effect was calibrated by setting the C 1*s* photoemission at 284.6 eV. The XPS depth profile of HfO_2_/TiO_2_/HfO_2_ on Pt- and TiN-coated Si was obtained by Ar ion etching. The electrical properties of the HfO_2_/TiO_2_/HfO_2_ trilayer-structure RRAM devices were measured by Keithely 4200 semiconductor characterization system on probe station (CasCade Summit 12000 B-M). A current compliance of 10 mA was imposed to protect the fabricated device units from damages of high current during set processes. The bias voltage was applied to the Pt top electrode with the grounded bottom electrodes of Pt or TiN.

## Results and Discussion

**Fig. 1:**
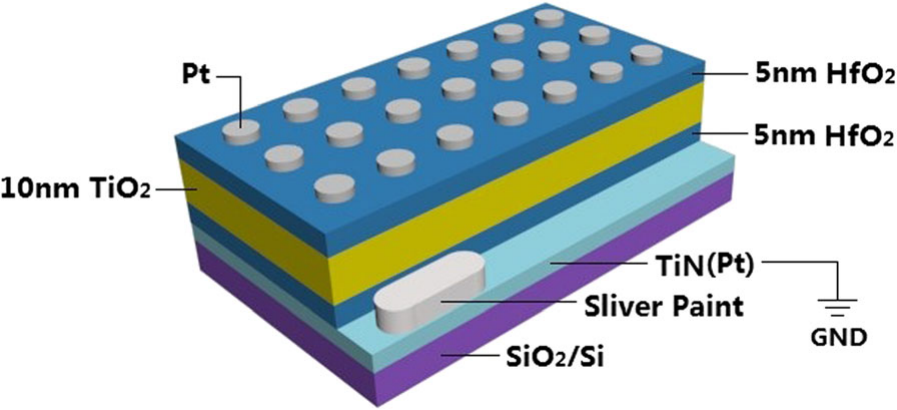
The schematic of the RRAM device of HfO_2_/TiO_2_/HfO_2_ trilayer-structure by ALD

The schematic of the RRAM device of HfO_2_/TiO_2_/HfO_2_ trilayer-structure by ALD is illustrated in Fig. 1. The surface morphology and roughness of the bottom electrodes and trilayer-structure of HfO_2_/TiO_2_/HfO_2_ on Pt- and TiN-coated Si have been examined. The Pt bottom electrode has smaller RMS value of 0.39 nm than PEALD-derived TiN of 0.87 nm. Hence, the sample of HfO_2_/TiO_2_/HfO_2_ on Pt-coated Si also exhibits relatively smoother surface with RMS of 0.68 nm than that on TiN-coated Si with 1.3 nm.

**Fig. 2:**
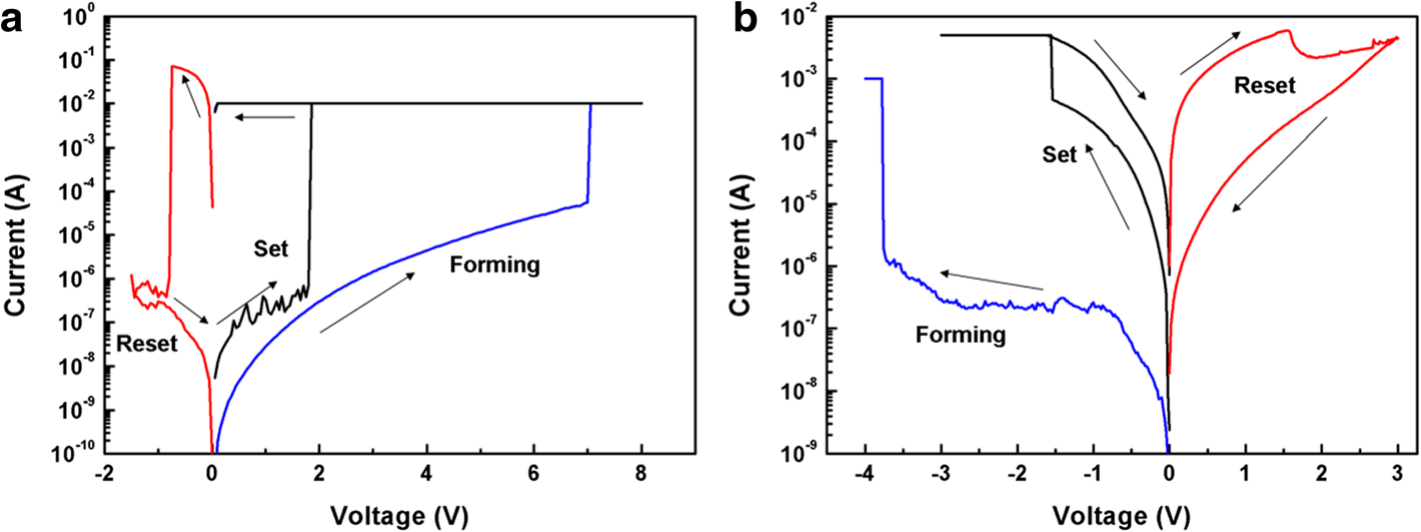
The typical bipolar resistive switching characteristics of the RRAM devices. **a** Pt/HfO_2_/TiO_2_/HfO_2_/Pt. **b** Pt/HfO_2_/TiO_2_/HfO_2_/TiN

The DC I-V curves of Pt/HfO_2_/TiO_2_/HfO_2_/Pt and Pt/HfO_2_/TiO_2_/HfO_2_/TiN devices containing the initial electroforming process are plotted in Fig. 2a, b, respectively, indicating a typical bipolar resistive switching characteristic. For almost all the samples, larger forming voltage is needed to form conductive filaments before the switching test. When applying a positive bias voltage on the Pt top electrode, the Pt/HfO_2_/TiO_2_/HfO_2_/Pt device unit shows a forming voltage of +7 V in Fig. 2a. With continuing the voltage sweeping, a reset voltage of −0.8 V is measured for unit cell from LRS to HRS and a set voltage of 2.0 V from HRS to LRS. The corresponding ratio of high and low resistances is about 10^5^. The electroforming and rest process can also be completed by applying a negative voltage of −11 V and a positive one of +4 V, respectively, which are much larger than the positive forming and negative reset voltages. Moreover, the device cell only switches from LRS to HRS for several cycles after the negative forming process and then fails to reset to HRS due to the irreversible breakdown (not shown here). In Fig. 2b, compared to that with symmetric Pt top and bottom electrodes, the tri-layer structure RRAM devices with asymmetric TiN bottom electrode and Pt top electrode show an opposite lower forming voltage of about −3.7 V, set voltage of −1.5 V, reset voltage of +1.5 V, and relatively smaller ratio of high and low resistances of 10^2^. When imposing the positive electroforming voltage, the RS phenomenon cannot be observed in the Pt/HfO_2_/TiO_2_/HfO_2_/TiN cell and the device is permanently broken down at +14 V without the following efficient reset from LRS to HRS at negative voltage (not shown here).

The trilayer-structure RRAM devices with symmetric Pt top/bottom electrodes and asymmetric TiN bottom electrode/Pt top electrodes exhibit such different electroforming polarity preference. The bottom electrode of chemically inert Pt or relatively active TiN seems to play a key role. The related reason on electrode-dependent electroforming polarity will be discussed later after considering the XPS depth profiles of trilayer structures of HfO_2_/TiO_2_/HfO_2_ on Pt- and TiN-coated Si.

**Fig. 3:**
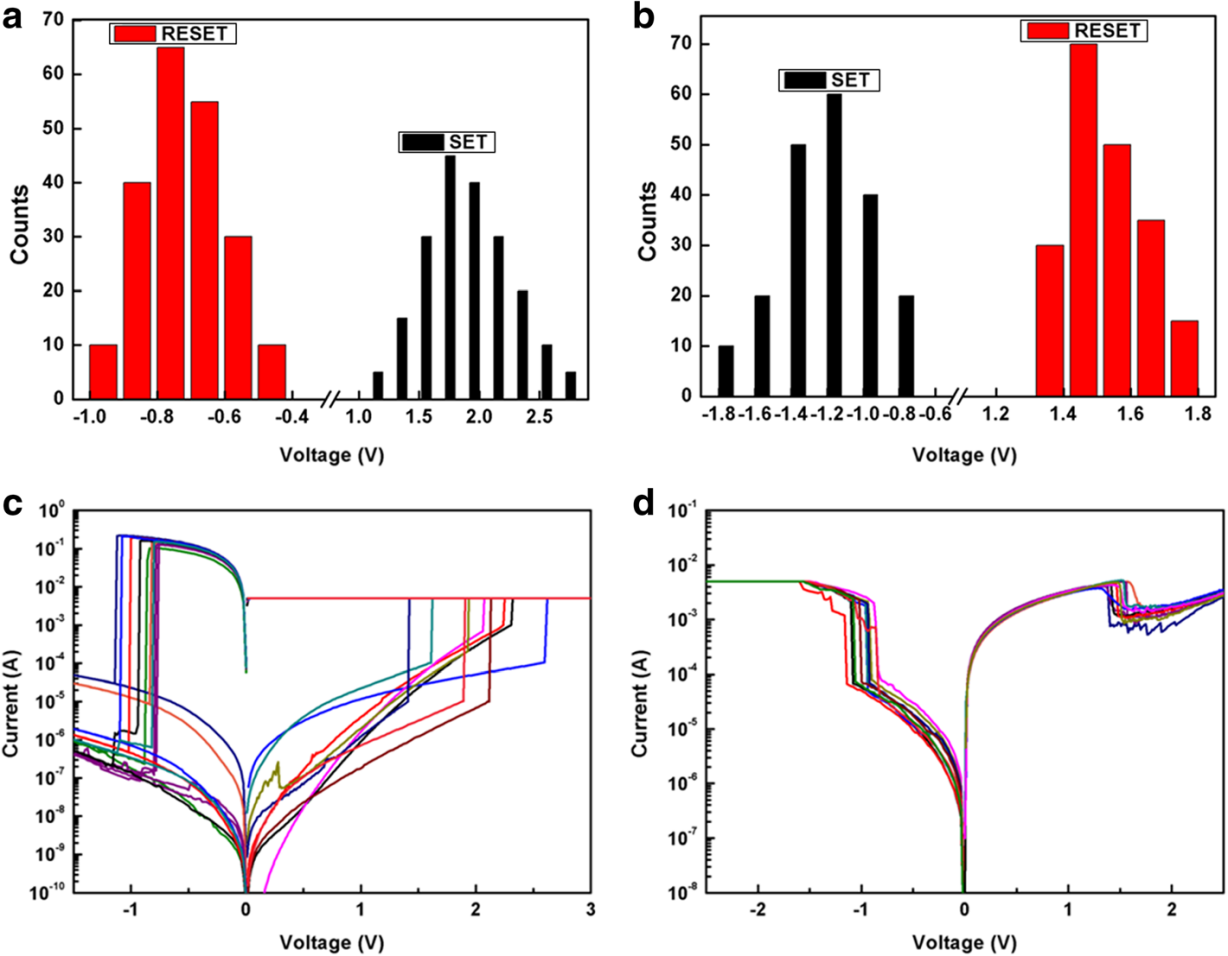
The statistical results of distribution of the set and reset voltages measured from a single device unit for 200 times tests. **a** Pt/HfO_2_/TiO_2_/HfO_2_/Pt. **b** Pt/HfO_2_/TiO_2_/HfO_2_/TiN. The I–V curves of 10 randomly selected device units. **c** Pt/HfO_2_/TiO_2_/HfO_2_/Pt. **d** Pt/HfO_2_/TiO_2_/HfO_2_/TiN

For high-density memory application, uniformity of RS parameters is very important. Figure 3a, b plots the statistical results of distribution of the set and reset voltages measured from a single device unit of the Pt/HfO_2_/TiO_2_/HfO_2_/Pt and Pt/HfO_2_/TiO_2_/HfO_2_/TiN for 200 times tests, respectively. Figure 3c, d records the I–V curves of 10 randomly selected device units of the Pt/HfO_2_/TiO_2_/HfO_2_/Pt and Pt/HfO_2_/TiO_2_/HfO_2_/TiN, respectively. The trilayer-structure RRAM device units with symmetric Pt top and bottom electrodes show a broad distribution for set voltage from 1.2 to 2.8 V and reset voltage from −0.5 to −1 V (Fig. 3a) and dispersive I–V curves (Fig. 3c). Whereas, the device units with asymmetric TiN bottom and Pt top electrodes display better RS behavior, such as relatively concentrated distribution of set voltage from −0.8 to −1.8 V and reset voltage from 1.3 to 1.8 V, and stable reproducibility in I–V curves (Fig. 3b, d). Simultaneously, compared to those on Pt-coated Si, different device units on TiN-coated Si also behave improved monodispersion in RS parameters, beneficial to RRAM practical applications.

**Fig. 4:**
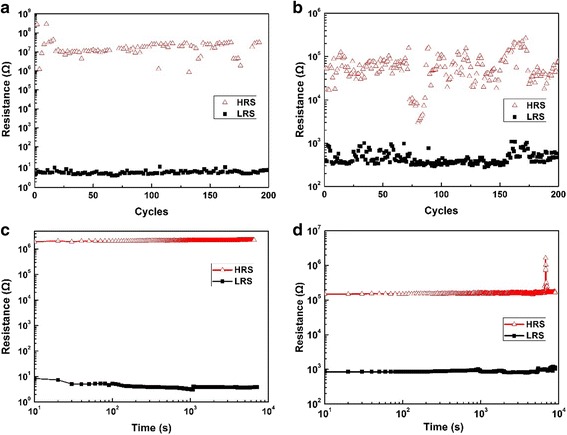
The endurance and retention characteristics of the device units. **a**, **c** Pt/HfO_2_/TiO_2_/HfO_2_/Pt. **b**, **d** Pt/HfO_2_/TiO_2_/HfO_2_/TiN.

The endurance and retention properties of the device units of Pt/HfO_2_/TiO_2_/HfO_2_/Pt and Pt/HfO_2_/TiO_2_/HfO_2_/TiN have been examined, as seen in Fig. 4a–d, respectively. In Pt/HfO_2_/TiO_2_/HfO_2_/Pt, the sweeping voltage was applied from 0 to 3 V for set and 0 to −1.5 V for reset. In Pt/HfO_2_/TiO_2_/HfO_2_/TiN, the sweeping voltage was applied from 0 to −2 V for set and 0 to 2 V for reset. The ON and OFF resistance values were read using 0.2 V at room temperature. The retention tests were measured at room temperature with the reading voltage of 0.2 V. After 200 program/erase cycles, Pt/HfO_2_/TiO_2_/HfO_2_/Pt device units show relatively stable resistance ratio of OFF/ON states above 10^5^ (Fig. 4a); however, the endurance characteristic of Pt/HfO_2_/TiO_2_/HfO_2_/TiN memory cells seem not to be as good as that of Pt/HfO_2_/TiO_2_/HfO_2_/Pt (Fig. 4b). The ON and OFF states in devices with Pt-TiN electrodes are not very steady with smaller resistance ratio of OFF/ON states of about 10^2^ during switching cycle test, especially significant HRS fluctuation than that of LRS. Based on the physical model in Ref. [12], the endurance will be improved by increasing the formation energy of oxygen vacancy and interface O^2−^ amount. We speculate one plausible explanation. In our previous work [14], excellent bipolar resistive switching properties of ALD-derived Al_2_O_3_/HfO_2_/Al_2_O_3_ trilayer-structures with asymmetric TiN bottom and Pt top electrodes have been demonstrated, including better switching endurance up to 10^3^ cycles with stable ON/OFF resistance ratio. Herein, we adopted HfO_2_/TiO_2_/HfO_2_ configure instead of Al_2_O_3_/HfO_2_/Al_2_O_3_. The metal ions in HfO_2_ and TiO_2_ have the same identical chemical valence of +4, leading to the less interface charged defects such as oxygen vacancies between two interfacial layers (ILs) of trilayer HfO_2_/TiO_2_/HfO_2_. Whereas, the metal ions in Al_2_O_3_ and HfO_2_ have different chemical valence of Al^3+^ and Hf^4+^, producing more interface charged defects of oxygen vacancies between two ILs of Al_2_O_3_/HfO_2_/Al_2_O_3_. It can be deduced that the interface O^2−^ amount by adding two ILs between Al_2_O_3_/HfO_2_/Al_2_O_3_ should be higher than between HfO_2_/TiO_2_/HfO_2_. In addition, the formation energy of oxygen vacancy in storage layer of Al_2_O_3_/HfO_2_/Al_2_O_3_ is also higher than that of HfO_2_/TiO_2_/HfO_2_ (formation energy of oxygen vacancy 7.08 eV (Al_2_O_3_), 6.53 eV (HfO_2_), and 4.35 eV (TiO_2_) [20, 21]). After considering these factors, the memory cell of Pt/HfO_2_/TiO_2_/HfO_2_/TiN exhibits endurance degeneration, compared to Pt/Al_2_O_3_/HfO_2_/Al_2_O_3_/TiN.

For Pt/HfO_2_/TiO_2_/HfO_2_/Pt and Pt/HfO_2_/TiO_2_/HfO_2_/TiN, the resistance level of the ON and OFF states has no evident changes after cumulative waiting time of 10^4^ s at room temperature in Fig. 4c–d, indicating a better retention property of both memory cells over 10 years based on the extrapolation method.

Pt/HfO_2_/TiO_2_/HfO_2_/TiN devices have much lower resistance ratio of OFF/ON states than Pt/HfO_2_/TiO_2_/HfO_2_/Pt, which can be attributed to the difference of the bottom electrodes in work function and conductivity. The work function of Pt and TiN bottom electrodes is 5.65 and 4.5 eV, respectively; hence, the interface barrier between TiN and underlying HfO_2_ is relatively lower in the Pt/HfO_2_/TiO_2_/HfO_2_/TiN device. During a reset process, the needed energy for electron to cross the barrier is also smaller. So Pt/HfO_2_/TiO_2_/HfO_2_/TiN manifests lower resistance value in OFF state than Pt/HfO_2_/TiO_2_/HfO_2_/Pt correspondingly. During a set process, RRAM device changes from HRS into LRS owing to the formation of the conducting channels. The Pt bottom electrode has higher conductivity than TiN bottom one; accordingly, the resistance value in ON state for Pt/HfO_2_/TiO_2_/HfO_2_/TiN is higher than that for Pt/HfO_2_/TiO_2_/HfO_2_/Pt. As a result, Pt/HfO_2_/TiO_2_/HfO_2_/TiN devices show a smaller resistance ratio of OFF/ON states of 10^2^. However, the resistance ratio of 10^2^ has already met the requirement of RRAM applications.

**Fig. 5:**
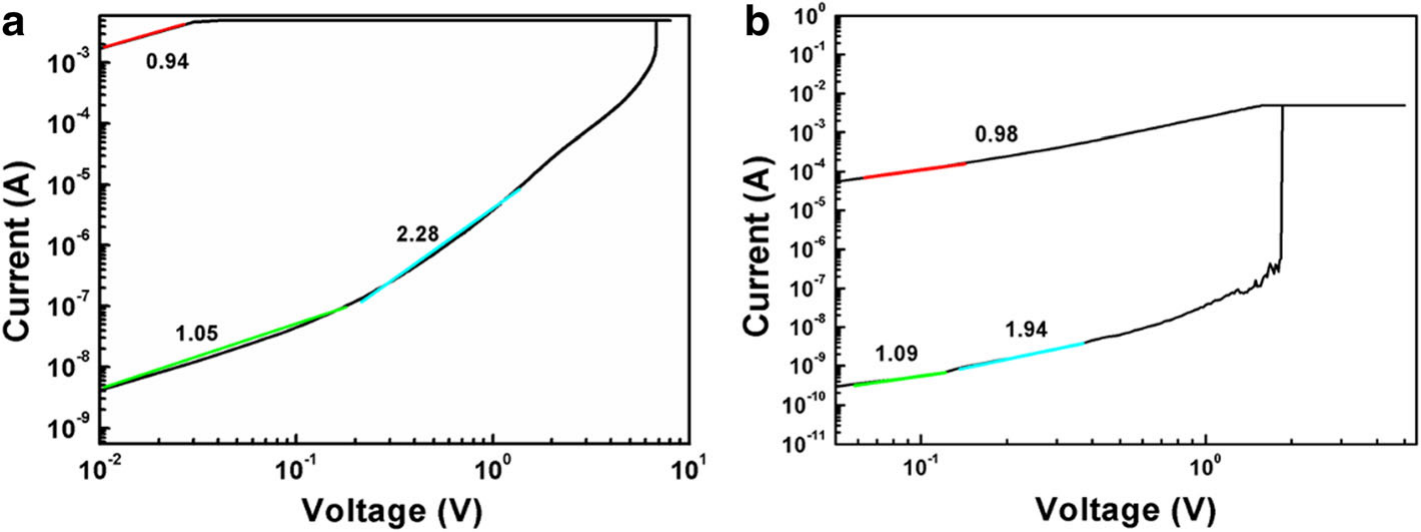
The typical I–V curves plotted in double-logarithmic scale of **a** Pt/HfO_2_/TiO_2_/HfO_2_/Pt and **b** Pt/HfO_2_/TiO_2_/HfO_2_/TiN

To clarify the conductive mechanism during resistive switching, the typical I–V curves are replotted in double-logarithmic scale. Figure 5a, b shows the linear fitting of the I–V curves for the voltage sweeping regions of Pt/HfO_2_/TiO_2_/HfO_2_/Pt and Pt/HfO_2_/TiO_2_/HfO_2_/TiN devices. Both memory cells behave similar conductive mechanism. When the device is switched to the LRS, the curves of log (I)-log (V) are linear with slope close to 1 (0.94 in Fig. 5a, 0.98 in Fig. 5b), indicating that the I–V at the LRS is dominated by the Ohmic law. The filament model of oxygen vacancy migration can be used to explain the switching behavior. For the HRS, at low-voltage region (absolute value <0.11 V), the I–V is dominated by the Ohmic law with the linear relationship of current and voltage (slope 1.05 in Fig. 5a, 1.09 in Fig. 5b). At higher voltage region (6.8 V > absolute value >0.11 V in Fig. 5a, 1.85 V > absolute value >0.11 V in Fig. 5b), the slope of the log (I)-log (V) lines are around 2 and the current is dependent of approximate square of applied voltage (I/V^2^). At critical voltage (absolute value 6.8 V in Fig. 5a, 1.85 V in Fig. 5b), a steep current rise suddenly appears with a very large slope. This result consists of three regions in HRS, basically obeying the typical trap-controlled space-charge-limited conduction (SCLC) injection [11].

In order to further understand the underlying resistive switching mechanism of HfO_2_/TiO_2_/HfO_2_ on Pt-coated and TiN-coated Si, we performed the XPS narrow scans and depth analyses on trilayer structures with symmetric and asymmetric bottom/top electrodes. XPS spectra were fitted with Gaussian-Lorentzian (G-L) functions after smart-type background subtraction.

**Fig. 6:**
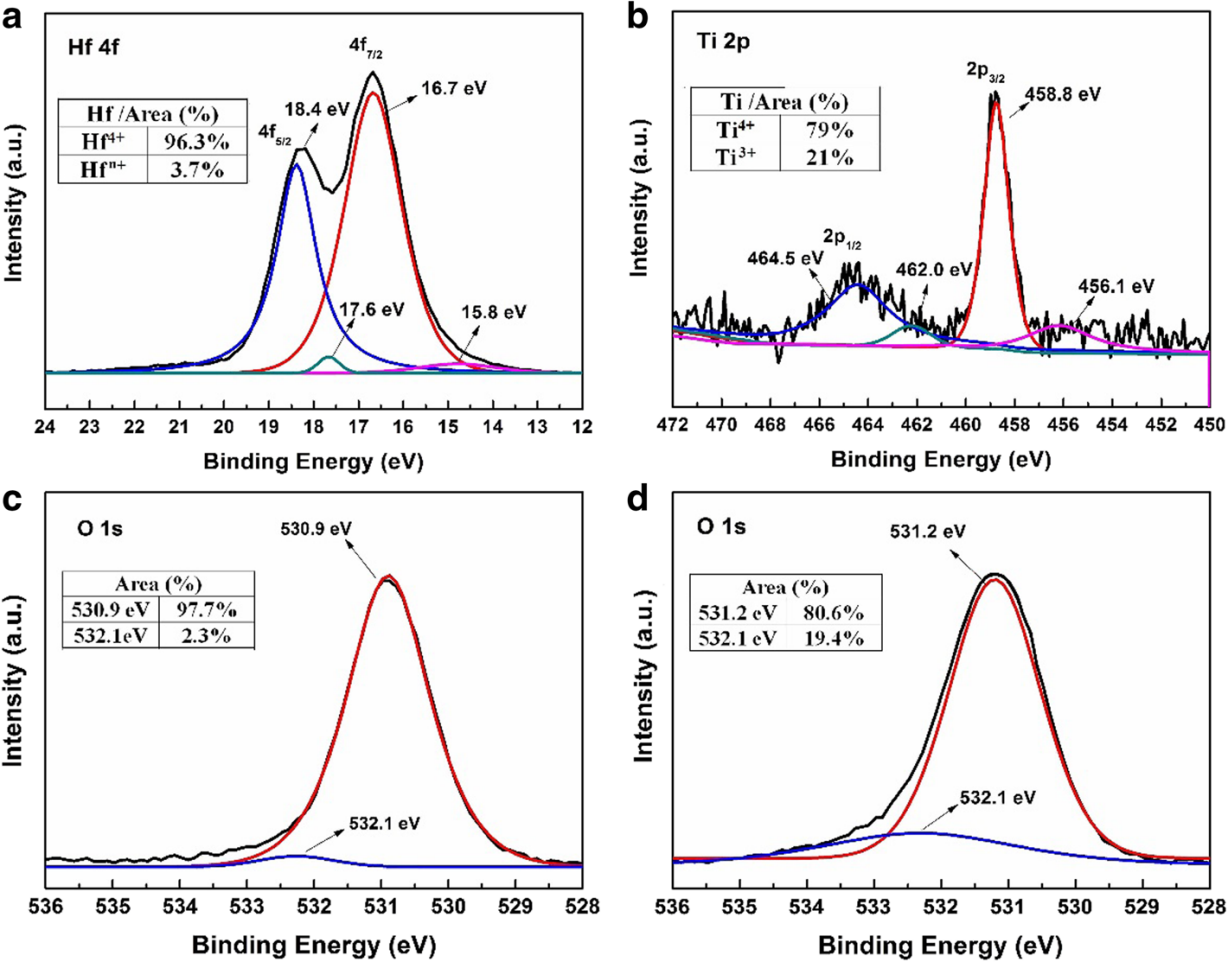
Narrow-scan XPS spectra from trilayer-structure of HfO_2_/TiO_2_/HfO_2_ on TiN-coated Si. **a** Hf 4f, **b** Ti 2*p* peaks of HfO_2_/TiO_2_/HfO_2._ O 1*s* peaks of **c** HfO_2_ and **d** TiO_2_ layers

Figure 6a–d illustrates the narrow-scan XPS spectra of Hf 4*f*, Ti 2*p*, and O 1*s* peaks in HfO_2_ and TiO_2_ layers on TiN-coated Si. The Hf 4*f* and Ti 2p spectra from HfO_2_ and TiO_2_ layers can be deconvoluted into two group peaks. The stronger Hf 4*f*_5/2_ and Hf 4*f*_7/2_ peaks at 18.4 and 16.7 eV with a spin–orbit splitting of 1.7 eV are assigned to Hf–O bonding (Hf^4+^) from HfO_2_ (Fig. 6a). The weaker spin–orbit doublet peaks at lower binding energy of 17.6 and 14.8 eV possibly result from the low-chemical valence state of Hf^*n*+^–O (*n* < 4), indicating the presence of oxygen vacancies in HfO_2_ layer. The calculated percentage concentration of Hf^*n*+^ (*n* < 4) is about 3.7% in Hf ions. In Fig. 6b, a stronger doublet corresponds to Ti 2*p*3/2 and 2*p*1/2 features at 458.8 and 464.5 eV, belonging to the Ti–O bonding (Ti^4+^) from TiO_2_. A weaker doublet locates at 456.1 and 462.0 eV, assigning to the Ti^3+^–O bonding. The calculated percentage concentration of Ti^3+^ is about 21% in Ti ions. This implies the existence of oxygen vacancies in TiO_2_ layer.

The O 1*s* spectra from HfO_2_ and TiO_2_ layers can also be deconvoluted into two peaks, as seen in Fig. 6c, d. The relatively lower binding energies of the O 1*s* peak at ~530.9 and 531.2 eV are assigned to Ti-O and Hf-O bonding in TiO_2_ and HfO_2_ layers, respectively, belonging to lattice oxygen without oxygen vacancies. Whereas the slightly higher energy of 532.1 eV in the O 1*s* spectra of Fig. 6c, d are ascribed to the oxygen atoms near oxygen vacancies in HfO_2_ and TiO_2_ layers based on the literature reports [22–25]. The relative oxygen vacancy concentration in the oxide layer can be roughly evaluated by calculating the area proportion of each peak [22, 23]. The calculated percentage concentration of oxygen vacancy in HfO_2_ and TiO_2_ layers is about 2.3 and 19.4%, respectively, in accord with the results of Hf^*n*+^ and Ti^3+^.

**Fig. 7:**
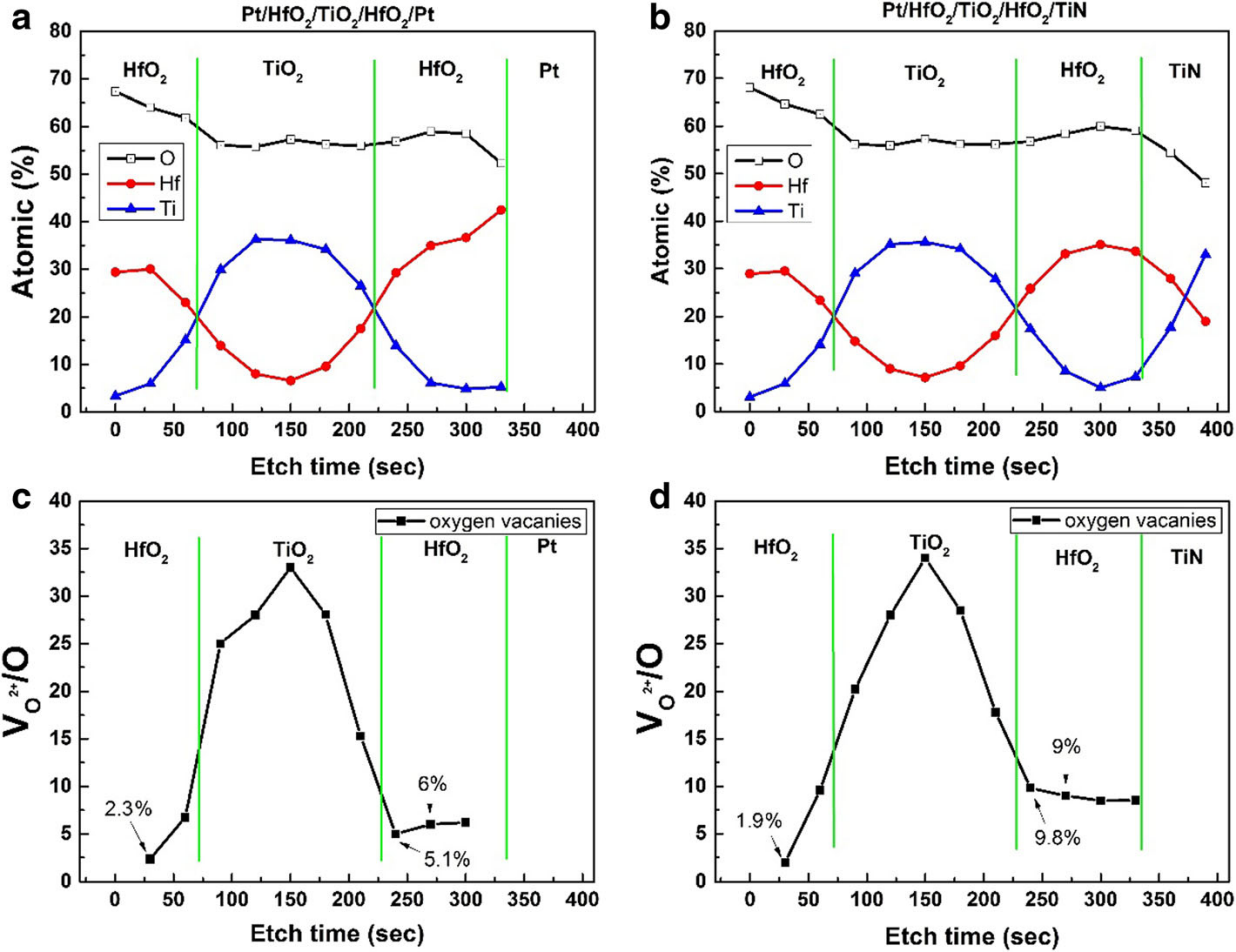
XPS depth profiles of HfO_2_/TiO_2_/HfO_2_ on Pt- and TiN-coated Si by Ar ion etching. **a** HfO_2_/TiO_2_/HfO_2_ on Pt-coated Si. **b** HfO_2_/TiO_2_/HfO_2_ on TiN-coated Si. The depth distribution of oxygen vacancy concentration (*V*_*O*_^2+^/O) determined from XPS spectra for HfO_2_/TiO_2_/HfO_2_ on Pt- and TiN-coated Si. **c** HfO_2_/TiO_2_/HfO_2_ on Pt-coated Si. **d** HfO_2_/TiO_2_/HfO_2_ on TiN-coated Si

Figure 7a, b shows the XPS depth profiles of HfO_2_/TiO_2_/HfO_2_ samples on Pt- and TiN-coated Si by Ar ion etching, respectively. The trilayer-structure of HfO_2_/TiO_2_/HfO_2_ on Pt- and TiN-coated Si can be recognized easily, although the significant interfacial diffusion between HfO_2_/TiO_2_ and HfO_2_/TiN has been observed. Usually the filament model of oxygen vacancy migration dominates the RS behavior in RRAM devices based on transition metal oxides [11]. However, simply increasing the oxygen vacancies content is not fully effective. How to effectively control the distribution of oxygen vacancy filaments is a key issue to finally improving the RS uniformity [20]. Lots of work has shown that usually a nonuniform distribution of oxygen vacancies is beneficial to the RS behaviors, including decreasing the forming voltage, improving the switching stability and endurance ability [1, 14, 20, 26–29]. An initial nonuniform distribution of oxygen vacancies in a storage oxide layer is often obtained by the use of a chemically active electrode with a relatively high oxygen affinity (e.g., Ta, Ti, Al, or TiN) or by deliberately introducing an oxygen vacancy-rich interfacial layer by interface engineering [1, 17, 30].

Figure 7c, d presents the distribution curves of oxygen vacancy concentration of HfO_2_/TiO_2_/HfO_2_ on Pt- and TiN-coated Si based on the above depth XPS profiles, respectively. The oxygen vacancy concentration was evaluated using the method mentioned above. It can be found that the highest oxygen vacancy concentration (~34%) appears in TiO_2_ intermediate layer. Another noteworthy feature is that the underlying HfO_2_ layer near Pt or TiN bottom electrodes has higher oxygen vacancy concentration than upper HfO_2_ layer (~2%). Besides, the oxygen vacancy concentration (~9%) of underlying HfO_2_ layer on TiN-coated Si is obviously higher than that (~6%) on Pt-coated Si.

**Fig. 8:**
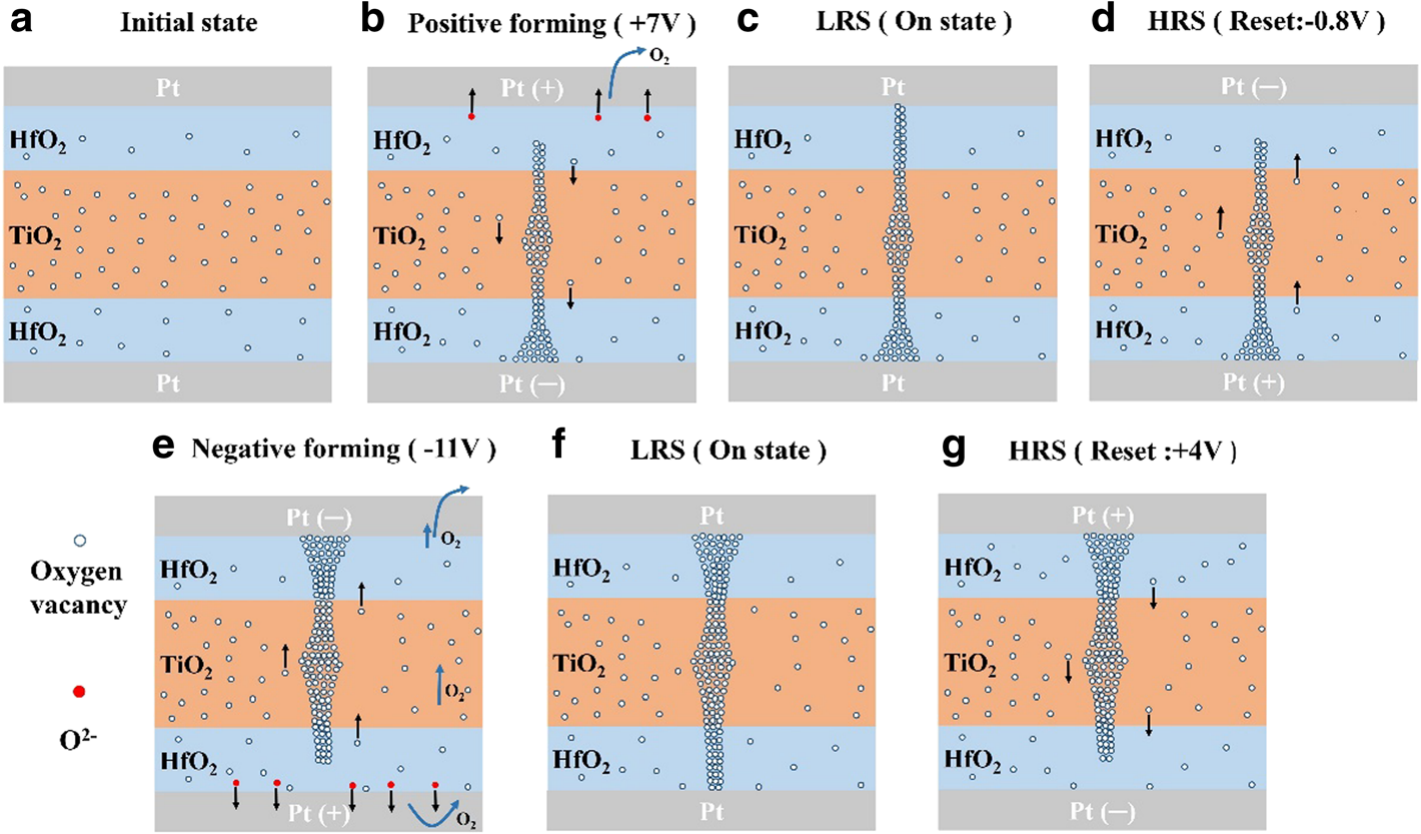
Schematic diagrams of electroforming and reset of trilayer-structure of HfO_2_/TiO_2_/HfO_2_ with symmetric Pt top and bottom electrodes. **a**–**d** Electroforming at positive voltage and reset at negative voltage. **e**–**g** Electroforming at negative voltage and reset at positive voltage

Figure 8 shows the schematic diagrams of electroforming and reset of Pt/HfO_2_/TiO_2_/HfO_2_/Pt at positive and negative bias voltages. For chemically inert Pt top and bottom electrodes, the bias polarity-dependent electroforming phenomena are related to the O_2_ gas released from the oxide layer at the anode as a product of electro-reduction. As known, during electroforming process, oxygen vacancies are created by high electric field and migrate to the cathode, forming localized conducting filaments in the oxide layer, i.e., *O*_*O*_→*V*_*O*_^*2+*^ + *O* ^*2−*^, *V*_*O*_^*2+*^ + 2*e* ^*−*^→*V*_*O*_ [12] (Fig. 8b, e). Simultaneously, O^2−^ ions drift towards the anode, releasing their charge and evolving O_2_ gas, i.e., *O* ^*2−*^→1/2*O*_*2*_ + 2*e* ^*−*^ (Fig. 8b, e), which possibly cause physical deformation of the cell and even formation of crack or hole [31]. When applying positive forming voltage to Pt top electrode, we postulate the O_2_ gas released above the upper HfO_2_ film easily escapes at the top electrode edge or through small nanopore in Pt top electrode (Fig. 8b), which causes weak physical deformation. Meanwhile, considering the relatively higher oxygen vacancy concentration in underlying HfO_2_ (~6%) nearby Pt bottom electrode than the upper HfO_2_ layer (~2.3%) (Fig. 7c), the conductive filament readily forms, leading to smaller positive forming voltage of +7 V. With reverse voltage of −0.8 V, the reverse reaction leads to the filament ruptures, and the device is easily switched back to the HRS state (Fig. 8d).

Conversely, with a negative forming voltage on the top electrode, oxygen gas forms under the underlying HfO_2_ layer and above Pt the bottom electrode (Fig. 8e). The O_2_ releasing becomes more difficult, which will impede the formation of conductive filaments. Moreover, because the oxygen vacancy concentration of the upper HfO_2_ layer (~2.3%) is lower than that of the underlying HfO_2_ (~6%) (Fig. 7c), the higher negative forming voltage of −11 V is needed to form filaments. When more O_2_ accumulates to a certain pressure under underlying HfO_2_, it possibly erupts from the mechanically weakest part of the thin films, leading to the hole in oxide films or separation between oxide films and the bottom electrode induced by bubble cracking. Although the device can be reset to HRS at applied +4 V bias (Fig. 8g), the memory cell is degraded after several switching cycles. In our samples, a part of top electrode was blown off after the forming process. Similar electroforming polarity preference scenario has also been observed in Pt/TiO_2−*x*_/Pt bipolar RRAM cells with various physical deformation evidences of the junctions [31, 32].

Pt/HfO_2_/TiO_2_/HfO_2_/TiN devices exhibit quite different electroforming polarity from Pt/HfO_2_/TiO_2_/HfO_2_/Pt. The LRS caused by electroforming can be observed at both negative and positive bias voltage; however, the effective reset from LRS to HRS can be achieved only at positive bias voltage in Pt/HfO_2_/TiO_2_/HfO_2_/TiN device. Similar electroforming preference with asymmetric Pt and TiN electrodes has been observed in some literatures with various storage oxide layers such as HfO_2_ [33, 34], TiO_2_ [35], ZrO_*x*_/HfO_*y*_ bilayer [22], and Al_2_O_3_/HfO_2_/Al_2_O_3_ trilayer [14]. However, the related explanations are divergent or lacking.

**Fig. 9:**
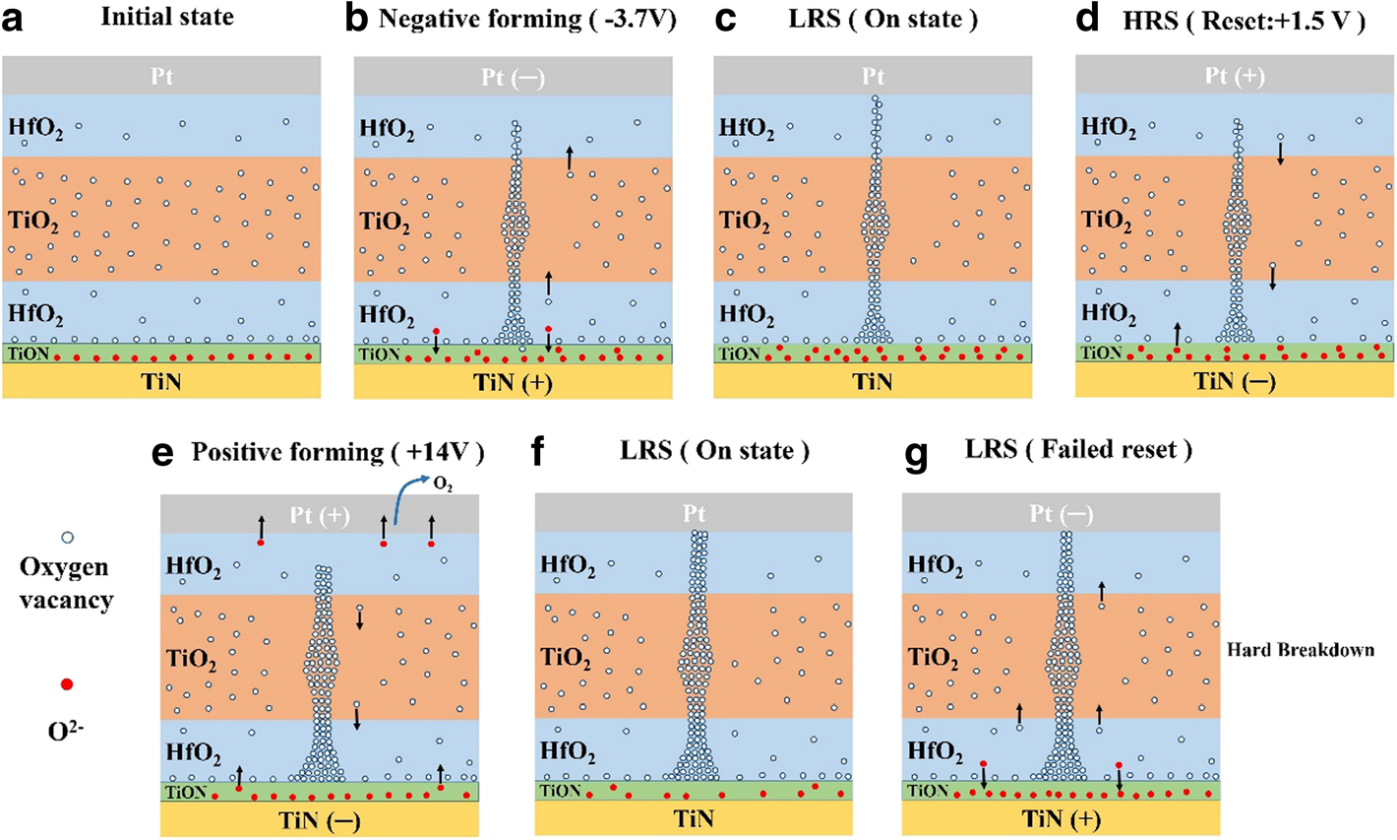
Schematic diagrams of electroforming and reset of trilayer-structure of HfO_2_/TiO_2_/HfO_2_ with asymmetric Pt top electrode and TiN bottom electrode. **a**–**d** Electroforming at negative voltage and reset at positive voltage. **e**–**g** Electroforming at positive voltage and reset at negative voltage

After considering the TiN electrode’s chemical activity with oxygen [1, 30, 36] and the nondistribution of oxygen vacancy concentration in trilayer-structure of HfO_2_/TiO_2_/HfO_2_ based on the XPS depth profiles (Fig. 7), a possible mechanism on electroforming polarity preference of Pt/HfO_2_/TiO_2_/HfO_2_/TiN cells is proposed. Figure 9 shows the schematic diagrams of electroforming and reset of Pt/HfO_2_/TiO_2_/HfO_2_/TiN at negative and positive bias voltages. The TiN bottom electrode plays a key role in the electroforming polarity. Kwak et al. reported that the relatively active TiN electrode would easily absorb oxygen ions from oxide films to form TiO_*x*_N_1−*x*_ (TiON) interfacial layer [36]. The severe oxygen diffusion of underlying HfO_2_ layer into TiN bottom electrode has been confirmed in our sample by the XPS depth profile (Fig. 7b). For Pt/HfO_2_/TiO_2_/HfO_2_/TiN device, the TiN electrode with high oxygen affinity [34, 36] produces a lot of oxygen vacancies in the underlying HfO_2_ layer near the TiN bottom electrode. The oxygen vacancies concentration of ~9% of underlying HfO_2_ layer is much higher than that of ~6% in the Pt/HfO_2_/TiO_2_/HfO_2_/Pt device.

For chemically inert Pt top electrode and relatively active TiN bottom electrode, when applying negative forming voltage, O^2−^ ions drift towards the TiN anode and are absorbed to form TiO_*x*_N_1−*x*_ layer (Fig. 9b), i.e., TiN + *x*O^2*−*^→TiO_*x*_N_*1−x*_ + *x*2*e*, avoiding the O_2_ release and possible damage to cells. The TiN electrode might act as a reservoir for oxygen ions drifting under an applied voltage [1, 36]. Simultaneously, due to the higher oxygen vacancy in the underlying HfO_2_ layer (~9%) and intermediate TiO_2_ layer (~10–34%) than the upper HfO_2_ layer (~1.9%), the migration of the rich oxygen vacancies from the underlying HfO_2_ layer through TiO_2_ layer towards the the upper HfO_2_ layer, directly producing conductive oxygen vacancy filaments with a smaller forming voltage of −3.7 V (Fig. 9b). When performing reverse reset process at +1.5 V, the bottom electrode of TiN layer acting as oxygen reservoir supplies oxygen ions to react with oxygen vacancy, i.e., O^2*−*^ + *V*_*O*_^2*+*^→*O*_*O*_ (lattice oxygen), which is beneficial to the reset operation from LRS to HRS with rupture of conductive filament (Fig. 9d). Above all, the oxygen reservoir effect of TiN bottom electrode contributes the forming voltage reduction as well as better set/reset voltage variation [1, 29, 36].

On the other hand, when imposing the positive forming voltage on top electrode Pt, the oxygen vacancies migrate from the upper HfO_2_ into the underlying HfO_2_ on TiN, accumulate in its vicinity, and develop into filaments. Owing to the lower oxygen concentration in the upper HfO_2_ (~1.9%) than the underlying HfO_2_ layer (~9%) and intermediate TiO_2_ layer (~10–34%), this leads to further increase of the oxygen vacancy concentration in underlying HfO_2_ and intermediate TiO_2_ layer. In the same time, due to slight oxygen existence in PEALD-derived TiN films (about 10%), some oxygen ions in TiN drift into underlying HfO_2_ layer to form insulate lattice oxygen with nearby oxygen vacancy, preventing from the growth of conductive filaments (Fig. 9e). Finally, when the bias voltage attains +14 V, the coarsening conductive filaments in oxides is formed. However, when a negative voltage is applied on the device, the conductive filament in trilayer structure is too large to be ruptured (Fig. 9g). Therefore, the device cannot switch to HRS by applying a negative voltage, indicating that an irreversible hard breakdown occurs in Pt/HfO_2_/TiO_2_/HfO_2_/TiN device.

Finally, on account of the fact that the inserted TiO_2_ layer stores more oxygen vacancies than HfO_2_ layer, the distribution of oxygen vacancies in trilayer structure is not uniform, especially in the two interfacial layers between HfO_2_/IL/TiO_2_/IL/HfO_2_, which might affect the growth position, direction, and overlapping of conductive filaments. The linkage or rupture of the conductive filaments corresponds to the set process from HRS to LRS or the reset process from LRS to HRS, respectively, which more easily happens in two interfacial layers. Further, the shape and position of the conductive filaments in HfO_2_ and TiO_2_ layers change less in the set and reset processes.

## Conclusions

In summary, RRAM devices based on trilayer-structure of Pt/HfO_2_/TiO_2_/HfO_2_/Pt and Pt/HfO_2_/TiO_2_/HfO_2_/TiN have been prepared by ALD. Both memory cells show typical bipolar resistive switching characteristics, and Ohmic and SCLC dominant conduction mechanisms in LRS and HRS, respectively. It is found that the bottom electrodes of Pt and TiN have great influence on the electroforming polarity preference, the ratio of high and low resistances and dispersion of the operating voltage of trilayer-structure memory cells. Compared to with symmetric Pt top/bottom electrodes, the RRAM cells with asymmetric Pt top/TiN bottom electrodes show smaller negative forming voltage of −3.7 V, relatively narrow distribution of the set/reset operation voltages and lower ratio of high and low resistances of 10^2^. The electrode-dependent electroforming polarity can be explained by considering electrodes’ chemical activity with oxygen, the related reactions at anode, and the nonuniform distribution of oxygen vacancy concentration in trilayer-structure of HfO_2_/TiO_2_/HfO_2_ on Pt- and TiN-coated Si. Furthermore, the TiN electrode as oxygen reservoir plays an important role in forming voltage reduction and better dispersion of RS parameters for Pt/HfO_2_/TiO_2_/HfO_2_/TiN devices. Considering the modulation effect of electrode and trilayer-structure on resistive switching performance, this work provides a new device design route for future RRAM applications.
